# Fermented fish products in South and Southeast Asian cuisine: indigenous technology processes, nutrient composition, and cultural significance

**DOI:** 10.1186/s42779-021-00109-0

**Published:** 2021-11-10

**Authors:** Yutika Narzary, Sandeep Das, Arvind Kumar Goyal, Su Shiung Lam, Hemen Sarma, Dolikajyoti Sharma

**Affiliations:** 1 Department of Botany, Bodoland University, Kokrajhar, BTR, Assam 783370 India; 2 Department of Biotechnology, Bodoland University, Kokrajhar, BTR, Assam 783370 India; 3 Pyrolysis Technology Research Group, Higher Institution Centre of Excellence (HICoE), Institute of Tropical Aquaculture and Fisheries (AKUATROP), Universiti Malaysia Terengganu, 21030 Kuala Nerus, Terengganu Malaysia; 4 Institutional Biotech Hub (IBT Hub), Department of Botany, Nanda Nath Saikia College, Titabar, Assam 785630 India; 5 Department of English, Gauhati University, Guwahati, Assam 781014 India

**Keywords:** Fish, Fermentation, Cultural significance, South and Southeast Asian cuisine, Food

## Abstract

The cleaner production of biomass into value-added products via microbial processes adds uniqueness in terms of food quality. The microbe-mediated traditional process for transforming biomass into food is a sustainable practice in Asian food industries. The 18 fermented fish products derived through this process as well as the associated micro-flora and nutritional composition have been focused. This review aims to update the process of green conversion biomass into value-added food products for a more sustainable future. Fish products are classified based on the substrate and source of the enzymes used in fermentation, which includes the three types of technology processing discussed. According to the findings, these fermented fish contain a plethora of beneficial microbiota, making them a valuable source of probiotics that may confer nutritional and health benefits. *Bacillus* (12 products), *Lactobacillus* (12 products), *Micrococcus* (9 products), and *Staphylococcus* (9 products) were the most common bacterial genera found in 18 fermented fish products. Consuming fermented fish products is beneficial to human health due to their high levels of carbohydrate, protein, fat, and lactic acid. However, biogenic amines, which are produced by certain bacteria as a by-product of their catabolic activity, are a significant potential hazard in traditionally fermented fish.

## Introduction

Fermentation is the microbial process of transforming food through the use of bacteria or fungus, as well as the enzymes produced by these microorganisms. This transformative ability of microbes has been utilized by people for production of fermented beverages and foods in order to preserve food and make the food more digestible with reduced harmfulness. Fermented foods involve controlling the microbial growth, which helps in the biotransformation of bioactive phenolics and volatile compounds into new edible forms with enhanced flavors and aromas [1, 2]. The microbial population in fermented food products deriving indigenously or from external 'starter cultures' causes chemical and textural changes to the food [3]. The microbial population in food can either cause spoilage of the food or produce a food product that is edible and safe for food, or there is a very thin line, determined by taste and flavor, between putrid and fermented food wherein the first one is undesirable, but the latter becomes a delicacy [4]. Fermented food has evolved with the development of human civilization since ancient times, partly sustaining the human population in the past and providing nutrition (calories, proteins, vitamins, minerals) at a reasonable cost. In particular, fermented fish is one of the most popular among the many different varieties of fermented food products. Although fermented food naturally contains live microorganisms (probiotics), the health benefits of their consumption are still uncertain [5].

**Fig. 1:**
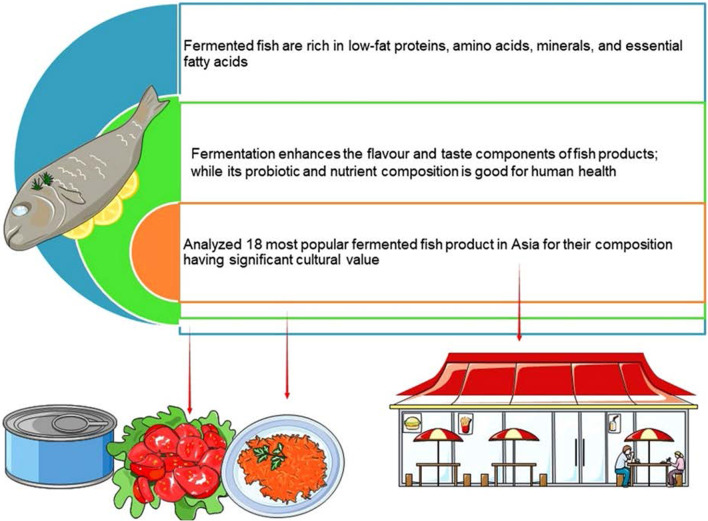
The microbe-mediated traditional process for transforming biomass into food is a sustainable practice in Asian food industries. The 18 major fermented fish products derived through this process as well as the associated micro-flora and nutritional composition have been focused

The usage of fermented fish as a dish in traditional culinary habits dates all the way back to ancient times in many societies throughout the world [6]. The earliest fermented fish sauce ever recorded is *garum* in classical Rome, which was prepared from the viscera and blood of mackerel [7]. Consigned to academic and cultural invisibility in food studies for the most part, this fish sauce has only recently come to be seen as having a striking similarity with fish sauces in Southeast Asia, from its umami taste and flavor and its ability to radically transform everyday dishes (Fig. 1) to its being cheap and commonly available [8]. In many cultures throughout history, people have developed diverse means to preserve food resources like fish during periods of scarcity following an abundant seasonal supply of fish [9]. The fish had to be preserved via variety of ways due to its perishable nature, including drying, salting, and fermenting [10].

**Fig. 2:**
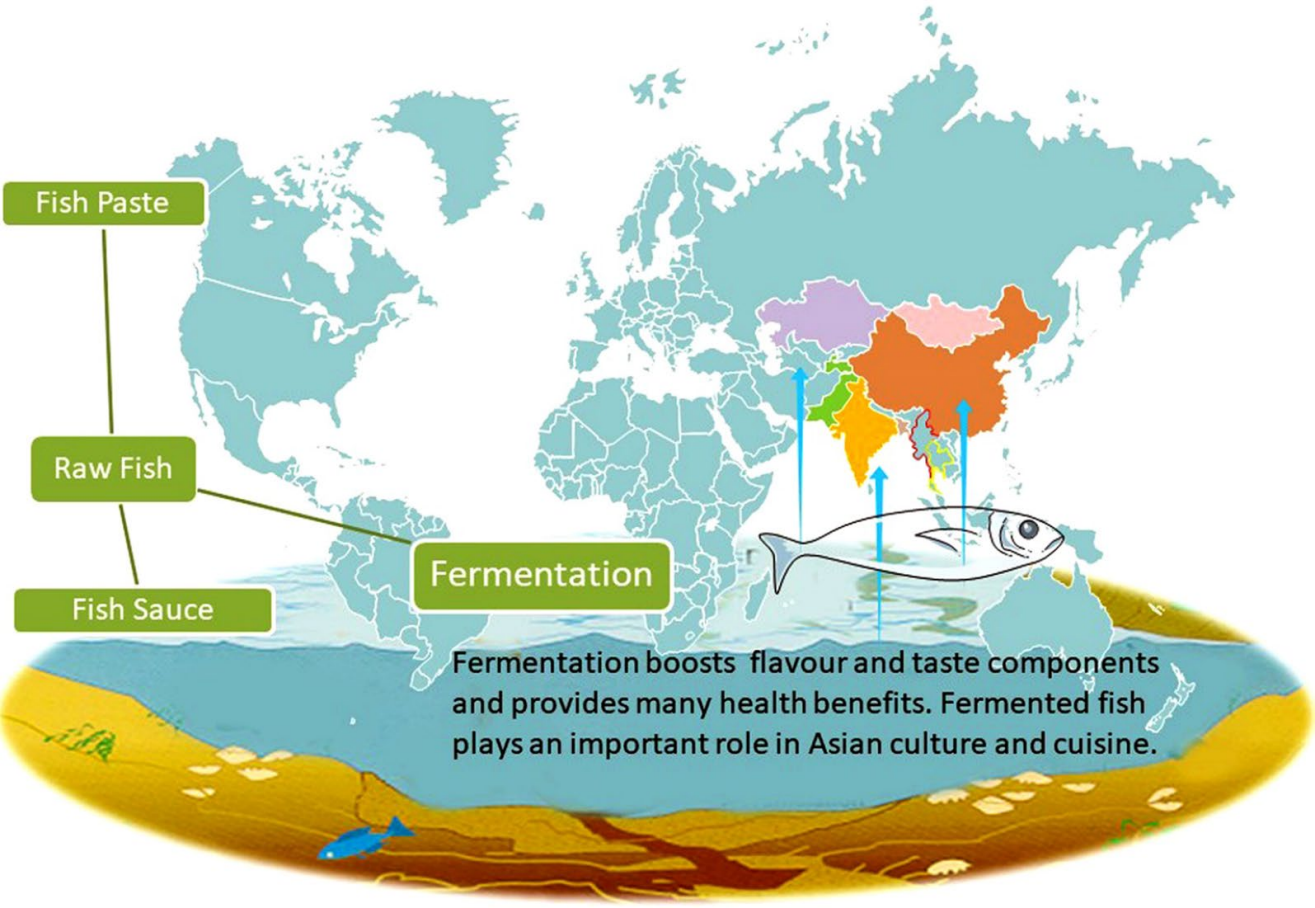
Global distribution of fermented food products

Apart from East, Southeast and South Asia (Fig. 2), countries in other parts of the world like the Middle East, Africa and the Scandinavian countries in Europe also produce and consume fermented fish products, like surstromming (fermented herring) and rakorret (fermented trout) in Scandinavia and fermented anchovy in France and Spain [11]. There is a considerable degree of artisanship associated with fermenting fish in all the communities where it is a part of their everyday life and culture. In Africa, there are three basic techniques, namely fermentation with salting and drying, fermentation and drying without salting, and fermentation with salting but no drying [12]. Moreover, while fermented fish pastes and fish sauces are more popular in Southeast Asia, fermented fish products in Africa are usually presented in the form of the whole fish or large chunks cut from it [13]. Again, Asian and African cultures mostly use the traditional methods of (spontaneous, i.e., without the addition of starter cultures) fermentation unlike those in Europe, North America, Australia, and New Zealand where the fermented food products are normally made with specific starter cultures, i.e., microbial inocula which are used to initiate fermentation [3].The inoculum used as a starter culture may be a single strain of bacteria or a consortium isolated from natural fermented food products.

Strategies such as limited drying, smoking, fermentation, and strategically utilizing substrates to stimulate the development of some beneficial microorganisms that inhibit the growth of pathogenic microbes are used to produce fish products in a traditional manner. The fermented fish products are a significant source of protein, forming an integral part of staple diet, and possessing great cultural significance in many Asian countries such as Korea, Japan, China, Thailand, Cambodia, Malaysia, Philippines, and Indonesia and in Asian foodways in general [14]. The term ‘foodways’ denotes the composite set of culinary practices, customs, beliefs, folklore, the intersection of ethnicity and food habits, material aspects of food, memories and nostalgia regarding food and culture, and so on, that defines the connection between a particular culture and its food habits and cuisine. In India, ranging from the Northeast to the South, it is an integral part of food culture [15]. Other South Asian countries like Sri Lanka, Bangladesh, Bhutan, and Nepal also have their own fermented fish products [16]. Fish fermentation is a small-scale industry and can provide direct employment to the people involved in it with very simple artisanal equipment [13]. According to a report by the FAO [12, 13], the market and production of fermented products in a country are influenced by the food habits of the people and market demands. In the developing countries of Southeast Asia, South Asia and Africa, there is a good market for fermented fish products [13, 17, 18].

In India, fermented fish is popular and various types of fermented fish products have been reported from its Northeastern region. *Matkashidal* is a widely known fermented fish that is economically viable in the Northeast Indian states of Manipur and Tripura [17]. Fermented fish is a staple food in South and Southeast Asian foodways, and the product has a large consumer base in the region. However, these products are typically produced at the family or village level using traditional methods in the region [19]. Some products like fish sauce and shrimp paste are, however, produced on an industrial scale and have good market potential. This has led to some nations embarking on improving the quality of such products, like the Cambodian government, which introduced strict industry standards (underlining certain specific characteristics like color and smell) in 2016 for the production of *prahok*, a type of fermented fish pastes and a national staple food (https://www.fareasternagriculture.com). This was due to the realization that the popularity of *prahok* among both resident and diasporic Cambodians made it necessary to monitor the health and safety issues related to the product.

In their study of the cultural contexts and significance of fermented fish and fish products in Southeast Asia, Ruddle and Ishige argued that this region should be considered a central point of origin of fermented fish products, as the freshwater fishes in the rice producing region (particularly in the rice fields) began to be fermented by the earliest indigenous populations [20]. The primary reasons for the fermentation of fish in this region since ancient times are the seasonal availability of fish and its scarcity during the long dry months. The abundance of water bodies and the fact that the cultivation of rice required a huge and consistent supply of water during its growing period (something that the region’s climate ensured) created a conducive environment for fish to flourish during the rainy season [20]. Furthermore, archaeological excavations and studies reveal that Southeast Asia was a hub of salt production (apart from more well-known archaeological sites in China) since prehistoric times [21]. These circumstances together compelled and facilitated the preservation of fish using fermentation methods, given the high degree of humidity in the region [20], leading to fermented fish and fish products dominating the food cultures in the region. One notable example is Northeastern Thailand where rice and fish are staple foods, while salt is the most commonly available preservative. As a result, these resources consequently define the food cultures in this region, in the sense that fish fermented using salt as an additive is a primary side dish that invariably accompanies and renders the staple diet of rice tasteful [22]. Due to the relative lack of economic security and modernized ways of life in many parts of Southeast Asia (especially in the rural areas like in Northeastern Thailand), producing and consuming fermented fish were (and still are) a cost-effective and accessible way of including proteins and minerals like calcium and phosphorus in the everyday diet [22]. In addition, fermented fish, with its umami taste, relieved the mundanity of the rice and vegetable diet of the local population [20, 22].

In the case of India’s Northeast, the climate, with its heavy rainfall and high humidity (making this region the wettest in India, and indeed, among the wettest regions in the world), made it impossible to dry the fish, while the sun shone, simply because there was not enough sunlight, unlike in the drier parts of India. The monsoon season from May to September naturally coincided with the fishing season, followed by a long period of scarcity of fresh fish in the region [23]. This necessitated an efficient and relatively faster method of fish preservation through fermenting, and it is thus that this part of India has a central presence in the fermented food map of India.

Another important factor is the similarity of the Northeast Indian communities and their ways of life with the larger Southeast Asian region and cultures. This owes partly to the strategic geographical location of the region, connecting, on the one hand, the Indian subcontinent with Southeast Asia. This contiguity as well as continuity extends to the shared food habits of the two regions (Northeast India and Southeast Asia) characterized by a centrality of rice and fish in Southeast Asian and Northeast Indian foodways. These shared sensibility and shared food preferences, again, embody a continuity of cultural habits and ways of life going back to ancient times that are also reflected, for example, through similarities in dress and clothing, as well as music and musical instruments (among other aspects). In fact, archaeological excavations have revealed that the Neolithic culture in Northeast India produced cord-marked pottery that is associated with the Neolithic cultures in Southeast Asia [24]. When placed within this framework, the cultural and gastronomical centrality of fermented fish does not merely symbolize the shared cultural identities of the various communities in the two regions but also points to the fact that foodways create a unique cultural map that is extremely fluid and extends across political boundaries.

Given their high cultural and economic significance, the study of fermented fish products gives an ample scope for further research. For these reasons, this review is organized to summarize the unique fermented fish products from Asia, their nutritional composition, cultural significance, and health implications. Furthermore, this article has focused on all Asian fermented fish cuisines. Because of the abundance of the literature on traditional Chinese cuisine, numerous examples of Chinese fermented food are readily available and recognized around the world. The database, however, contains only a limited amount of information about other Asian countries. Due to a lack of fermented fish composition analysis in the rest of Asia, we focused on fermented fish from South and Southeast Asia to narrow the information gap.

## Sources of review of related literature and studies

A search was conducted using keywords that included the terms ‘nutrition,’ ‘biochemistry,’ and ‘microorganisms,’ as well as fermented fish, fish sauce, and fish paste, on a variety of bibliographic databases such as ‘Pub Med,’ ‘Scopus,’ and ‘Google Books.’

**Fig. 3:**
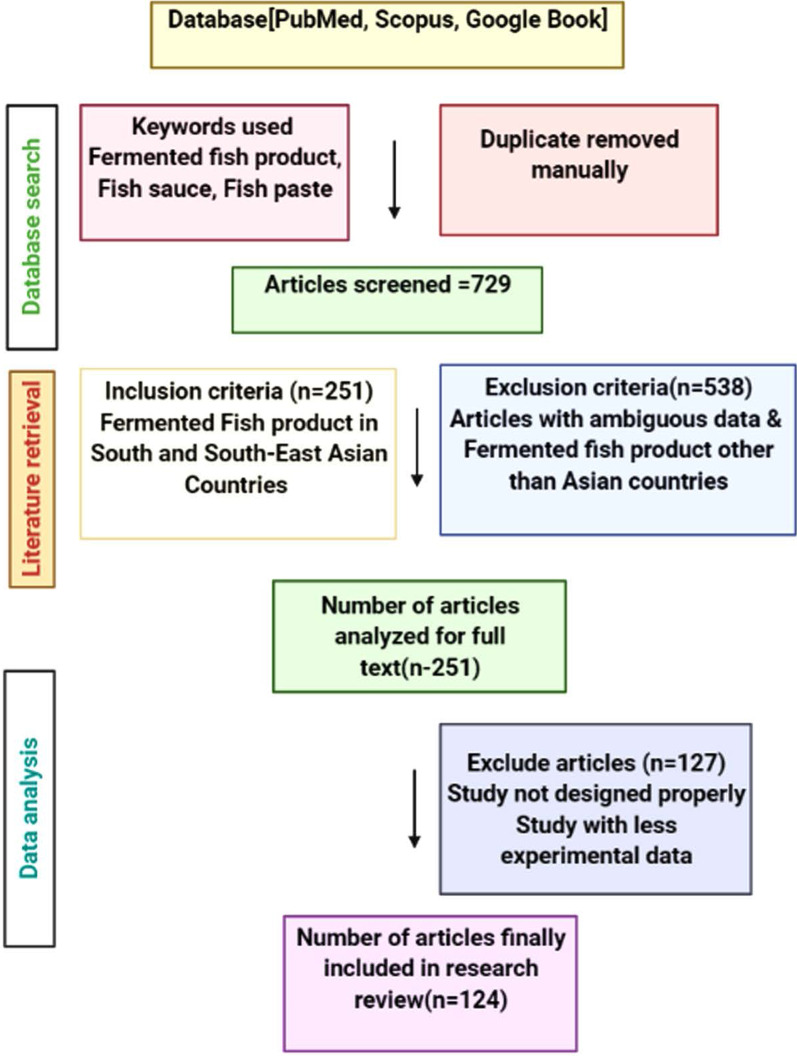
The research design used in this food composition analysis is depicted in a flowchart

To ensure that the scientific names of the fish species corresponded to the accepted names, synonyms, families, and author citations, we searched a web-based database. Of the 729 articles analyzed for this purpose, only those that contained relevant information were screened for data extraction. In addition, 28 books chapters (available in Google Books) that are not in the ‘Pub Med and Scopus’ database but are important for this food composition analysis have been included in this study. The methodology and criteria used in the study are depicted in detail in Fig. 3.

## Fermentation technologies and types of associated fish products

Throughout history, different cultures have adopted a range of techniques to slow down the bacterial and enzymatic degradation of fish including salting, drying, smoking, and fermentation. Fermentation is one of the most ancient and significant traditional methods of food preservation. It entails the production of alcohol, lactic acid, and carbon dioxide from food sugars, primarily through the action of microorganisms and enzymes, resulting in foods with distinct quality characteristics such as aroma, taste, shelf life, texture, and nutritional value that differ significantly from the raw material [25]. Fish sauces and fish pastes are the most popular fermented fish products in many Asian countries and are regarded as indispensable condiments in these culinary cultures. Fish sauce is produced due to changes in physical and chemical characteristics brought about by microbial activity in high salt concentrations and low oxygen levels [26]. Another form of fermented fish product is one where the fish retains its solid form and does not get reduced to a paste or a liquid form. Based on the country and its traditional practices, fish sauce is made by combining fish and salt in a 2:1 to 6:1 ratio [14].

Traditional and non-traditional fish products are classified in different ways according to their respective mechanism of processing, substrates used, salt concentration, and the form of the final product. Saisithi [27] has divided such processes and their resultant fermented fish products into three categories. The first is a purely traditional and natural process based on enzymes present in the fish flesh and entrails with salt as seasoning. In the second category, microbial enzymes and salt are added in the form of 'starter cultures' on the flesh of fish and entrails. The third category consists of non-traditional products manufactured by artificially induced accelerated fermentation. Rattagool [28] classified traditional fermented fish items in Southeast Asia based on the substrates used or added during the fermentation processes, such as salt and carbohydrate. Saisithi [29] proposed a classification system for traditional fermented fish products based on the substrate and source of the enzymes used in the fermentation that comprises three groups: (1) where carbohydrates are not a part of the fermentation and the product is the result of the activity of the bacterial enzyme already present in the fish and salt mixture; (2) where carbohydrates are present in the fish and carbohydrate mixture used in the fermentation process; and (3) where a 'starter culture' is used that brings about the fermentation of the carbohydrates. The most usual process, however, is the first one where the fermentation is brought about by the enzymes present in fish.

**Fig. 4:**
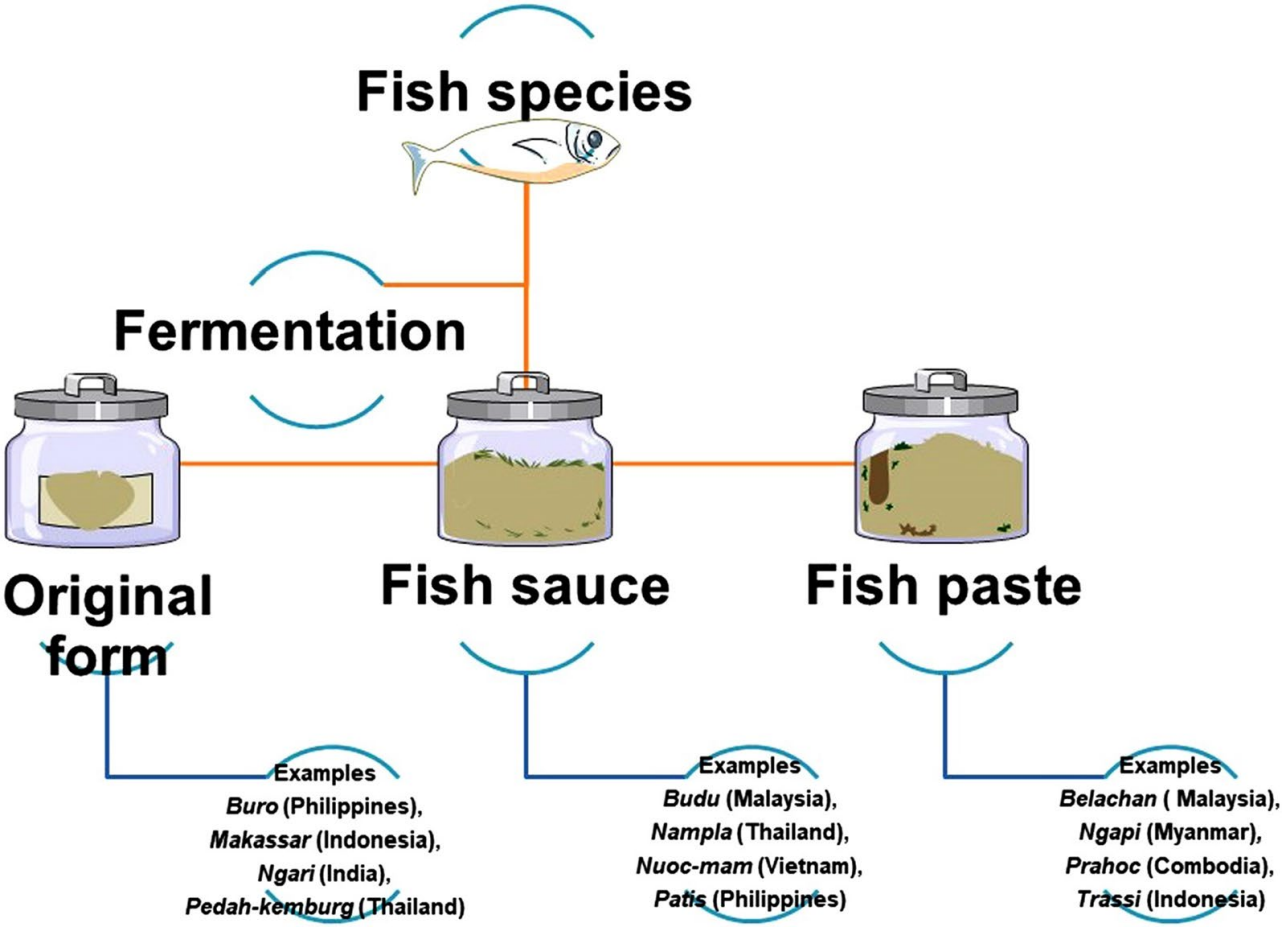
Fermented fish products in various forms that are considered a delicacy in Asian cuisine (Rao [31]; Beddows [14])

Ruddle and Ishige [30] gave a well-defined generic classification and methods of preparation of fermented fish of East Asia. They classified fermented fish into two groups. In one, freshwater fish was used for fermentation, and the other consisted of marine finfish and shellfish. Normally, traditional fermented fish products are salted products with different salt concentrations. Accordingly, fermented fish can also be classified into highly salted products with 20% salt, low salt products with 6–8% salt, and products without any added salt [30]. Fermented fish products are also categorized according to the form retained by the final product [14, 31]. The main types of fermented fish according to the form are the following: (a) Fermented fish products retaining their original form which are often salted, like *buro* (Philippines), *makassar* (Indonesia), *pedah-kemburg* (Thailand), *makassar* (Indonesia), *ngari*(India), Colombo cured mackerel (India); (b) products that contain fish that have been reduced to a liquid, e.g., *patis* (Philippines), *budu* (Malaysia) *nampla* (Thailand), and *nuoc-mam* (Vietnam); and (c) Products in paste form, e.g., *ngapi* (Myanmar), *bagoong* (Philippines), *prahok* (Cambodia), *trasi* (Indonesia), and *belacan* (Malaysia) (Fig. 4).

Fermented fish is significant traditionally and economically in East and Southeast Asian countries. Because of its unique flavor and taste, its popularity in these countries is far reaching [32]. It is a common food in northern Laos, Cambodia, southern Myanmar, northeastern Thailand, and the region of Luzon in the Philippines. All three forms of fermented fish products are prevalent in these countries. Again, in many fermented fish products of Asia, *koji* (*Aspergillus* sp.) is added to enhance the fermentation [33, 34]. The Asian climate has a higher humidity and temperature that is optimal for filamentous fungi like Aspergillus, Penicillium, Rhizopus, Mucor to thrive, leading to their wide use in fermentation processes in various parts of the continent [35]

In Thailand, well-known fermented fish products are *budu, kapi*, and *prala* [36]. *Budu* is an intermediate between fish sauce and fermented fish paste and has a colloidal consistency, and *prala* is fermented fish paste where rice bran is added as a carbohydrate source [27]. *Kapi*, on the other hand, is a fermented shrimp paste using salt in a ratio of 5 (shrimp):1 (salt), that is then dried and compacted [37]. The technology used for the preparation of these products may be similar, but the additives are different depending on the product, with ingredients such as rice and fruits being used in many places. The use of an additive facilitates the growth of lactic acid bacteria, lowering the pH of the solution. Sometimes, similar products may also have diverse names in different localities of Thailand.

The traditional fermented fish products of *Malaysia* are*: pakesan* prepared from freshwater fish mixed with tamarind and salt, *belacan* (shrimp paste), *cincaluk, budu, and kikapikan*. In the Philippines, fermented fish products produced with lactic acid bacteria are consumed in the Central Luzon area [27]. *Bagoong* (fish paste), *balao-balao* (fermented shrimps), *burongisda* (fermented rice and fish), *bagoong alamang* (shrimp paste), and *paite* (fish sauce) are some of the most common and popular fermented fish products in the Philippines. It was reported that many fishes, including herring and anchovy, and shellfish, such as shrimp, are used for the preparation of *bagoong* by mixing salt with well-drained samples. *Bagoong* has a characteristic salty taste, faintly cheese-like odor, and prepared from fish or shrimp. The fish sauce is called *paite* and obtained from thoroughly fermented *bagoong*. *Balao-balao* is a dish made with cooked rice and shrimp (*Penaeus indicus* or *Macrobrachium* sp.), while *burongisda* is made with freshwater fish and rice [38].

The most popular fermented fish products in Indonesia are* pedah* (fatty, partly dried, salty fish), *trasi* (shrimp paste), *bekasang*, and *bekasam* (freshwater fish that has been fermented), *jambal roti* (marine fish that has been fermented and then dried and salted), *budu*, *cincaluk*, *naniura*, *petis*, *picungan*, *pudu*, *rusip*, and *tukai* [39, 40]. Fish products that have undergone fermentation (*terasi, peda, and kecapikan*) account for less than 2% of all fermented fish products processed [40].

Myanmar’s traditional fermented fish *ngapi* made from salted fish or shrimp is considered the cornerstone of Burmese traditional food [4]. *Nuocmam* is a fish sauce consumed by a huge majority in Vietnam. It is prepared from small fish and shrimps. The fish are layered with salt and kept for fermentation. The supernatant liquor is decanted after it has aged to produce high-quality *nuocmam* [41]*.* During the fermentation process of fish sauce, the fish tissue is extensively liquefied, and the resulting fluid portion is collected as the sauce [42].

Fermented fish sauce, like *yu-lu* which is commonly consumed in China as a condiment, is a popular and indispensable seasoning in Chinese cuisine. Anchovies (or other small marine fish) are allowed to ferment by adding salt at the level of 30–40%, salt/fish (*w/w*) in this product. The period of fermentation is 12–18 months [43]. In Japan, the fermented fish is called *shiokara* (preserves the whole or partial of the fish shape), and *shiokara* paste (yielded by pounding or grating). When, along with fish, additives like salt, vegetables, or steamed rice are used, then it is known as *narezushi*. *Shiokara* and *shiokara* pastes are mostly used as condiments and side dishes [20, 44].

Korea also possesses a rich dietary tradition of fermented food and fish. Some of the major fermented fish products of Korea are *jeotkal, eo-ganjang,* and *sikhae* [45]*.* In the context of India, the practice of pit-curing is widespread, particularly along the country's coastlines. The salted fish are buried in pits lined with mats for a period spanning from a few days to two weeks or more in this curing method. The finished product is partially dry and has a distinct flavor and taste that is popular in the eastern region of Tamil Nadu, India [46].

The ‘Colombo cured’ method is a prevalent practice in Southern India and Sri Lanka for the preparation of fermented fish. Typically, this method involves mixing mackerel fish and salt in a 3:1 ratio in a concrete tank, along with dried tamarind. Improvements in the quality are obtained if 5% acetic acid is added in place of tamarind [3, 47].

The fish curing industry has a large presence in India, but paradoxically, it is also one that lacks mainstream commercial visibility though it is popular as a local cottage industry in the northeastern and coastal parts of India [48]. Fermented fish products, again, are quite prevalent in North East India and West Bengal. It is interesting to note that many such products, like *shidal* and *ngari*, do not use salt [23]. Salt, which has a long and contentious colonial history in India, was introduced in the Northeastern region by the British people. In place of salt, sometimes a local alkali called *khar* is used, which helps in alkaline fermentation (*napham*, *nichow* and *nakham *). The fermented fish pastes prepared in this region are pounded with various herbaceous plants like taro and allowed to age inside bamboo stems. Bamboo is abundant in this region, which has the highest diversity of bamboo species in India [49]. The use of bamboo stems and leaves in cooking is a widespread practice in Northeast Indian communities that finds its way even into aspects of folk cultures and studies of the region. Cooking food or processing it in bamboo stems imparts a unique flavor to the food. Thus, the fermented foods found in this region are unique and one-of-a-kind to a great extent due to the use of bamboo in their processing. Two types of fermented fish products have been reported in North East India: one that retains its solid form after fermentation and the other in a paste form. They are used as condiments or with other vegetables for their texture and flavor. The local people also associate these products with some health benefits. North East India's fermented fish products are diverse.

**Fig. 5:**
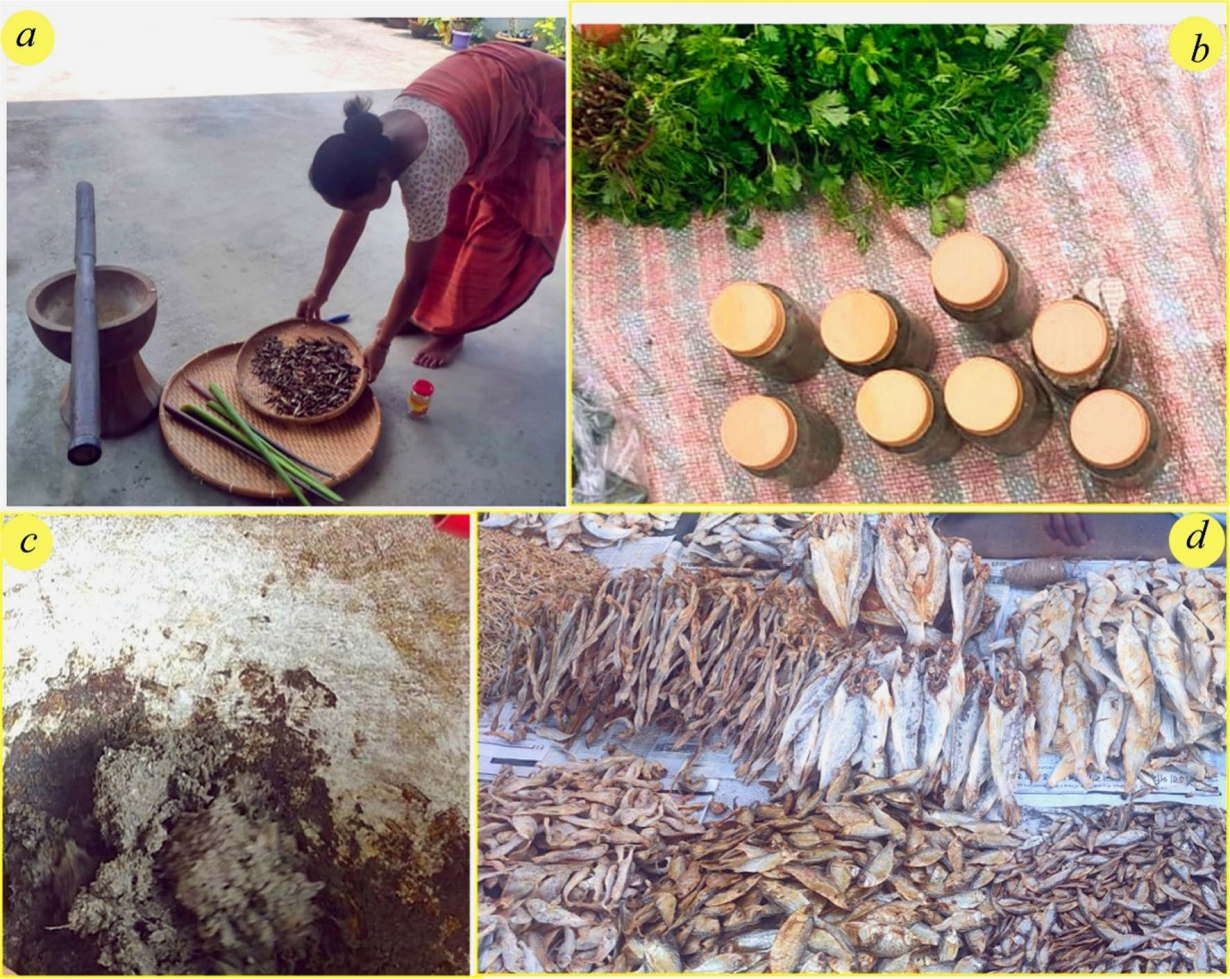
**a** A Bodo woman arranging the ingredients to make *Napham*. *Napham* is made traditionally to this day using mortar and pestle, and all the ingredients are available locally. Both the process and the final product are integral to the articulation Bodo cultural identity. **b** Jars of *Napham* sold in a local market in Assam. **c** The texture and consistency of *Napham* paste. **d** *Shidal* and other fermented fish sold in a local market in Assam

*Shidal* is a popular fermented fish product found in different states of Northeast India (Fig. 5). It is prepared from *Puntius* sp. (generally *Puntius sophore*) and *Stipinna phasa* and is known by different names across five states of Northeast India, namely Assam, Mizoram, Arunachal Pradesh, Nagaland and Tripura [23]. *Ngari* is a traditional Manipuri dry, unsalted fermented fish product made solely from sun-dried *Puntius sophore* Ham [50]. *Tungtap* is traditional fermented fish in Meghalaya which is prepared from *Puntius* sp. and *Danio* sp. [51]. *Hentak, numsing, sukakomacha, sukati, napham, nichow, nakham* are some other fermented fish products which are prepared by different ethnic communities found in Northeast India [36, 52]. *Lonailish* is salted and prepared exclusively from Indian shad (*Tenualosa ilisha*) popular in both Bangladesh and parts of India bordering Bangladesh [53].

## Microbiota of fermented fish products

Microorganisms are an integral part of all fermented food products. *Lactobacillus* and other microorganisms in fermented food have gained the status of GRAS (Generally Recognized As Safe) which has enhanced the commercial importance of fermented food products due to the beneficial physicochemical properties endowed by these microbes [54]. A broad spectrum of microorganisms has been reported from different fermented fishery products of Asia (Table 1, Fig. 4). The diversity of microbial strains is quite interesting, and their presence is not uniform. In the present study, *Pseudomonas aeruginosa* and *Escherichia coli* have been reported in only one product (*Pedah*). Similarly, *Staphylococcus aureus* was discovered in five different products, including *pedah, belacan, ngari, tungtap*, and *shidal*. However, we discovered that the most prevalent genera are *Lactobacillus, Staphylococcus*, and *Bacillus*, which are found in 12 of the 18 products included in this study. *Micrococcus* and *Staphylococcus* were found in 9 products, making them the second most prevalent bacterial genera in fermented fish. Furthermore, we discovered that *Pseudomonas*, *Flavobacterium*, and *Brevibacterium* were less dominant strains, with their presence recorded in five, three, and two products, respectively.

**Table 1:** Microorganisms found in various fermented fish items documented in the literature

Sl no	Fermented fish product	Country	Micro-organisms
1	*Pedah*	Indonesia	*Pseudomonas aeruginosa, Staphylococcus aureus, Escherichia coli, and Bacillus cereus* [98];* Lactobacillus plantarum, Pediococcus pentosaceus* [99]
2	*Jeotgal*	Korea	*Bacillus, Brevibacterium, Flavobacterium, Micrococcus, Pediococcus, Pseudomonas, and Staphylococcus, Halobacterium, Halomonas* [66, 100]*. Sarcina, Leuconostoc, Paenibacillus tyraminigenes, Bacillus subtilis, Leuconostoc mesenteroides, Pseudomonas halophilus, and Sarcinaitoralis* [101]. *Achromobacter, Corynebacterium, Micrococcus, Pediococcus, Alcaligenes, Aeromonas, and Moraxella* [102], *Salinivibrio, Staphylococcus, and Tetragenococcus/Halanaerobium* [103]
3	*Budu*	Thailand and Malaysia	*Lactobacillus plantarum* LP1, LP2*, Staphylococcus arlettae* SA1*, Saccharomyces cerevisiae* SC3 *and Candida glabrata* CG2 [104] *Acinetobacter* sp., *Flavobacterium* sp*., Cytophaga* sp., *Halobacterium* sp., *Micrococcus* sp., *Staphylococcus* sp. *and Corynebacterium* sp; *Lactobacillus* sp *and Staphylococcus* sp*.* [105]
4	*Nam-pla*	Thailand	*Micrococcus* sp., *Pediococcus* sp., *Bacillus* sp., *Halobacterium* sp. *(Lactobacillus* sp., *Pseudomonas* sp*., Corynebacterium* sp., *Streptococcus* sp*.,Sarcina* sp., [29]; *Tetragenococcus halophilus, Bacillus subtilis and Staphylococcus* sp. [106]; *Bacillus cereus, Bacillus circulans, Bacillus licheniformis and B. megaterium* [107]
5	*Plaa-som*	Thailand	*Lactococcus garvieae, Weissella cibaria, Streptococcus bovis, Pediococcus pentosaceus, Lactobacillus plantarum, and Lactobacillus fermentum* [108], *Pediococcus cerevisiae, Lactobacillus brevis, Staphylococcus* sp*.,and Bacillus* sp. [109]
6	*Belacan*	Malaysia	*Bacillus, Pediococcus, Lactobacillus, Micrococcus, Sarcina, Clostridium, Brevibacterium, Flavobacterium, Bacillus cereus, Staphylococcus aureus, and Corynebacterium* [40]
7	*Pekasam*	Malaysia	It was found that commercial *pekasam* is contaminated with *Listeria ivanovii, L. innocua, L. seeligeri, L. welshimeri, and L. denitrificans* due to the lack of proper handling [110]
8	*Trasi (Shrimp paste)*	Indonesia	*Staphylococcus, Bacillus, Proteus* were found to be present in *trasi* and *Salmonella, Vibrio, E. Coli* and *Clostridium* were absent [40]
9	*Bekasam*	Indonesia	*Enterobacter, Micrococcus, Lactobacillus, Moraxella, Eriococcids., Pseudomonas, Staphylococcus, Streptococcus* [111]
10	*Bagoong and Patis*	Philippines	*Bacillus* sp., *Pediococcus* sp.* Leuconostoc mesenteroides*, *Lactobacillus plantarum* [112]
11	*Balao-balao*	Philippines	*Leuconostoc mesenteroides, P. cerevisiae, Lactobacillus plantarum* [113]
12	*Sikhae*	Korea	*Paenibacillus halophilus* (in mixed fermentation), *Micrococcus* sp., *Halobacterium sp., Halococcus* sp., *Bacillus* sp. *Leuconostoc mesenteroides, Lactobacillus plantarum* [101]
13	*Ngapi*	Myanmar	Bacterial population found in Hmyin are *Aeromonas liquefaciens, Alcaligenes faecalis, Bacillus alvel, Bacillus badius, Bacillus brevis, Bacillus cereus, Bacillus circulans, Bacillus polymyxa, Bacillus firmus, Bacillus laterosporus, Bacillus lentus, Bacillus macerans, Bacillus pantothenticus, Bacillus stearothermophilus. Bacillus subtilis, Corynebacterium hoffmanni, Kurthia* sp., *Lactobacillus, streptobacterium, Pseudomonas fluorescens*, *Serratia marcescens and Staphylococcus epidermidis* [58]; *Tetragenococcus halophilus, Staphylococcus epidermidis, Tetragenococcus muriaticus, Halanaerobium fermentans, Tetragenococcus muriaticus, Clostridium *sensu*, Clostridium botulinum, Halanaerobium fermentans* [114]
14	*Ngari*	India	*Lactobacillus plantarum, Bacillus subtilis and Bacillus pumilus*[115] *Aspergillus, Cladosporium, Penicillium, Micrococcus, Staphylococcus and Bacillus* species identified were *Bacillus coagulans, Bacillus pumilus, Bacillus subtilis and Bacillus pantothenticus. Streptococci* and *Staphylococcus aureus* were also detected [116]
15	*Hentak*	India	*Enterococcus faecium, Lactobacillus fructosus, Lactobacillus amylophilus,* and *Bacillus subtilis* [115]
16	*Tungtap*	India	*Bacillus pumilus, Micrococcus, Bacillus cereus, Staphylococcus aureus, Candida, Saccharomycopsis, Enterococcus, Lactobacillus coryniformis, Lactobacillus puhozi, Lactobacillus fructosus, Lactobacillus plantarum, Lactococcus lactis, Lactobacillus amylophilus* *Bacillus subtilis* [115]
17	*Shidal*	India and Bangladesh	*Staphylococcus aureus, Micrococcus* sp., *Bacillus* sp., *E. Coli* [117]
18	*Shottsuru (Fish sauce)*	Japan	*Aerococcus viridans, Halobacterium* sp., halotolerant and halophilic yeasts, *Micrococcus, Corynebacterium, Streptococcus, Bacillus* in 2.5% NaCl and *Halococcus, Halobacterium, Vibrionaceae* and *Streptococcus* in 20% NaCl [118]

*Suanyu* is a fermented fish product popular among the indigenous people of Southwest China. Suanyu's dominant bacteria were *Lactobacillus, Tetragenococcus*, and *Weissella*, contributing 53.99%t, 35.60%, and 4.10%, respectively [55]. In *budu,* the strains of bacteria isolated were *Tetragenococcus halophilus, Bacillus licheniformis, Staphylococcus piscifermentans,* and *Bacillus subtilis* [56, 57]. In *pedah*, *Lactobacillus* sp. and *Staphylococcus* sp. were reported (Table 1). In *ngapi*, *Bacillus* sp. *Staphylococcus* sp., *Staphylococcus aureus*, *Micrococcus* sp., and *Pseudomonas* sp. were reported [58]. In *nuoc mam*, a fermented fish from Vietnam, microbial species like *Staphylococcus* sp., *Bacillus* sp., *Pseudomonas* sp., *Pediococcus* sp., *Lactobacillus* sp., *Streptococcus* sp., *Halobacterium cutirubrum, Halobacterium salinarum* was isolated [26, 59].

These studies suggest that *Bacillus* sp., *Staphylococcus* sp., *Lactobacillus* sp., *Micrococcus* sp. have a specific role in fish fermentation. In all these fermented fish products mentioned above, the raw materials used and the manufacturing processes differ, and these factors influence the type and number of microorganisms present in them. In some cases, the addition of certain microorganisms as ‘starter cultures’ leads to the acceleration in fermentation time resulting in a better product [61].

The addition of proteolytic enzymes of *Aspergillus oryzae* and *Aspergillus niger* hastened the production of *bagoong* and *patis*, respectively [62]. *Staphylococcus* sp.SK1-1–5 and *Virgibacillus* sp. SK 33 strains, when used as ‘starter cultures,‘ increase the desired volatile compounds in the fish sauce by inducing a higher production of proteinases. It has been claimed that *Tetragenococcus halophilus* improves the flavor of fish sauce [63] by producing volatile compounds like benzaldehyde, 2 &3- methylbutanal, and 2-methylpropanol that give the desired flavor to the fish sauce. Fungi like *Aspergillus luchuensis* have been attributed with generating a superior flavor due to their proteolytic and lipolytic activities in low salted fermented fish in a study by Kim [64]**.** Thus, endogenous enzymes found in a wide spectrum of microbes contained in fermented fish products can impart desirable organoleptic properties and prolong their shelf life. The balanced metabolites present in fermented fish can determine the preferable flavor and texture [65, 66]. Furthermore, the microorganisms also degrade the anti-nutritional components and produce metabolites with less toxic in nature. Histamine, for example, is a toxic amine found in a variety of fermented fish products that has serious health consequences. In a study by Kuda [67], *Tetragenococcus halophilus* isolated from *nukazu* had a suppressive effect on histamine accumulated in salted fermented fish***.**** Weissella* strains from *plaa-som* produced antibacterial compounds and folate [68]. Table 1 and Fig. 5 present information on the microbial components of Asian fermented fish products. In the analysis and review of 18 different fermented fish products of South and Southeast Asia, it was found that *Bacillus*, *Lactobacillus*, *Micrococcus* and *Staphylococcus* were the most predominant.

## Biochemical characterization of fermented fish in Asia

In the process of fish fermentation, biotransformation of proteins and fatty acids takes place as these are broken down into simpler compounds by microbes or the enzymes produced by them. The fish also contain indigenous enzymes that can bring about the breakdown of fish protein that cause the textural change in the final product. South Asian fermented fish products are alkaline [69]. In alkaline fish products, the hydrolysis of proteins into peptides and amino acids takes place naturally, releasing ammonia. The release of ammonia raises the final product's pH. The alkaline pH and ammonia in these products control the growth of a few dominant bacteria, which allows the anaerobic breakdown of proteins that release amine compounds. The high pH, free ammonia, and the rapid growth of essential microorganisms do not allow the sustenance of those bacteria that may spoil the product. Thus, the product is very well preserved and stable when dried [70, 71]. The majority of alkaline fermentations occur spontaneously in mixed bacterial cultures, with *Bacillus subtilis* predominating [70]. The physico-chemical characterization of various types of fermented fish is dependent on a variety of factors, including the type of processing fish used, the percentage of salt in the product, the number of days of fermentation, and the presence of microorganisms in the product*.* The biochemical characteristics of different fermented fish found in Asia are summarized in Table 2.

**Table 2:** Biochemical composition of different fermented fish products of Asia

	Fermented product	Country	Fish species	Process	Nutrient composition	References
1	*Pedah*	Indonesia	Mackerel (*Rastrelliger neglectus)* as raw material	Salting is carried out using a 1:3 salt to fish ratio and fermentation time 2–3 months	Parameter amount in 100 g sample Moisture content: 46 (g) Protein content (g): 28 g Fat content (g): 4 g Ca: 174 mg P: 316 mg Fe: 3.1 mg	[119]
2	*Jeotgal*	Korea	Various *Engraulis japonicus (anchovy)* and /or *Ammodytes* sp.	Salt at the level of 20–30%	Moisture content: 67.6% Crude protein content: 7.4% Crude fat content: 1.1% Carbohydrate: 0.01% pH: 6	[66]
3	*Budu*	Thailand and Malaysia	Sea water or brackish water fish *Stolephorus indicus, Clupeoides* sp., *Sardinella* sp., *Anchoviella commersonii,* and *Anchoviella indica*	Salt: 19.4–20.6%	Moisture content: 45–60.2% Crude protein content: 12.1–13.2% Crude fat content: 1.3–1.8% Fiber: 0.2–0.4% Ash: 21.6–23.2% Acidity: 0.4–0.9% pH: 5.4–6.5	[120, 121]
4	*Nam-pla*	Thailand	*Anchoviella* sp., *Stolephorus* sp., *Rastrelliger* sp., *Cirrhinus* sp.	5–12 months fermentation time 5:1 fish: salt	Crude protein content: 4.62% Moisture: 32.26% Fat content: 0.80% Lactic acid: 0.32% pH: 5.3–6.7	[120, 121]
5	*Plaa-som*	Thailand	Snakehead fish, salt, palm syrup and sometimes roasted rice, Barbs	Salt: 5.23–9.14%	Crude protein content: 16.08–18.94% Moisture: 62.2–67.72% Fat content: 0.71–3.20% Lactic acid: NF pH: 4.3–5.3%	[121]
6	*Belacan*	Malaysia	Small shrimps of Acetes and Mysid varieties	1–2 months fermentation; salt: 13–25.3%	Crude protein content: 28.7–40% Moisture: 27–40% Fat content: 1.4–2.6% Ca: 2.0–3.4% Fe: 0.02% pH: 7.2–7.8%	[122]
7	Pekasam	Malaysia	Freshwater fish, tamarind and salt, marine fish is also used	2–4 weeks; salt: 10–16%	Crude protein content: 15–25% Moisture: 57–73% Fat content: 3.0–8.0% Lactic acid: 0.1–0.4% Ash: 6–14% pH: 4.5–6.1%	[122]
8	*Trasi* (Shrimp paste)	Indonesia	Shrimp	7 days salt: 23%	Crude protein content: 25.89% Moisture: 12.13% Fat content: 3.8% Ash: 32–46% pH: 6.35–6.51%	[40]
9	*Bekasam*	Indonesia	Freshwater fish; common carp (*Cyprinus carpio* Linn.) cooked rice and sticky rice	Fermentation time: 1 week; salt 5.72%	Crude protein content: 4.81% Moisture: 66.95% Fat content: 14.25% Ash: 5.76% pH: 6.35–6.51%	[119]
10	*Bagoong and Patis*	Philippines	*Stolephorus* sp., *Sardinella fimbriata and Decapterus* sp.	1: 3 to 2: 7 (salt: fish) Bagoong takes smaller time and prolonged fermentation of Bagoong results in Patis fish sauce	Total nitrogen: 2.26% Formol nitrogen: 1.31% Ammonia nitrogen: 0.31% Sodium chloride: 26.10% pH: 5.85% lactic acid content: 0.84%	[123]
11	*Balao-balao*	Philippines	(*Penaeus indicus* or *Macrobrachium* sp.), adding salt (20% based on the shrimp weight)	1: 4.8 (weight of unsalted shrimp to rice). Salt (3%) is mixed thoroughly before the mixture is packed in wide mouth glass jars. The mixture is allowed to ferment for 7 to 10 days at 28 °C	pH: 7.52–3.80 Volatile acid (as acetic acid): 0.03–0.1% Titratable acidity (as lactic acid): 0.12–2.0%	[123]
12	*Sikhae*	Korea	Lactic acid fermented fish or clam meat in mixture of glutinous rice and salt	2–3 weeks of fermentation; low salt 2%	Organic acids- lactic acid, succinic acid and acetic acid NH2–N: 127 mg/100 g NH2–N: 127 mg/100 g VBN: 27.0 mg/100 g pH 5.0–4.6	[102, 124]
13	*Ngapi*	Myanmar	Fish, shrimp	Pounding or grinding of fish or shrimp with some salt; salt 22%	Moisture: 7.6% Protein: 41.9% Fat: 8.4% NaCl: 37.95% Free ammonia: 0.17% Ash: 3.45% Sand: 0.5%	[58]
14	*Ngari*	India	*Puntius sophore* and *Puntius ticto*	5–6 months	Moisture: 36.03% Total lipid: 13.34% Ash: 5.49 pH: 6.74 Water soluble nitrogen: 3.62% Free fatty acid-97.60%	[116]
15	*Hentak*	India	*Esomus danricus* and *Alocasia macrorrhizos*	Fermented fish paste, used as condiment or curry	Moisture: 20.9% Crude protein: 52.86% Crude fat: 17.10% Ash: 8.10% Free fatty acid (as oleic acid): 39.6% pH: 6.6 Non protein nitrogen: 1.1% Total volatile base nitrogen: 99.85 mg%	[117]
16	*Shidal*	India and Bangladesh	*Puntius sophore* and *Setipinna phasa*	Semi-fermented, unsalted product; 4–6 months fermentation; curry/pickle	Protein: 27.2–38.35% pH: 6.1–6.2 Moisture content: 33.44–37.52%	[125]
17	*Shottsuru* (Fish sauce)	Japan	Anchovy, opossum shrimp, salt	–	Salt conc.: 26.2–30.4%, pH: 4.54–5.56 Total nitrogen: 301–1598 mg/100 ml VBN: 36.2–170 mg/100 m	[126]
18	*Tungtap*	India	*Puntius* sp. and *Danio* sp.	Dried fish are kept inside the vessel to ferment	NR	[51]

*NR* not reported

The presence of amino acids, both essential and non-essential, in fermented fish products has been widely reported [27, 72]. Glutamic acid and aspartic acid were the most abundant amino acids in *rusip*, an Indonesian fermented fish. Histidine, serine, glycine, arginine, threonine, tyrosine, methionine, alanine, and valine were among the other amino acids found in *rusip* [73]. Glutamic acid contributed to the production of the characteristic flavor and aroma in this fermented fish product [74]. In *hatahata-zushi*, a Japanese fermented fish, the fatty acid profile consisted of elaidic acid (C18:1n-9), palmitic acid (C16:0), docosahexaenoic acid (C22:6n-3), eicosapentaenoic acid (C20:5n-3), and PalC16:1n-7 [75]. The dominant fatty acids reported in *ngari* were oleic acid (23.58%), palmitic acid (C16:0), and vaccenic acid (C18:1n-7) [76]. Fatty acids reported in *Hentak* were stearic acid (C18:0), palmitoleic acid (C16:1n-5), and oleic acid [76]. There were 62 volatile compounds identified and reported in Chinese traditional fermented shrimp, including, aldehydes, alcohols,, acids, ethers, esters, pyrazines, hydrocarbons, ketones, and phenols [77]. The literature suggests that the production of flavor and texture of fermentation fish depend on the amino acids and fatty acids that have been formed after microbial decomposition.

## Health and safety concerns of fermented fish

Fermentation of fish imparts desirable organoleptic characteristics and improves mineral bioavailability in the product [78]. Fermented fish contains significantly lesser amounts of fatty acid compared to fresh fish [79]. Fermented fish oil has been found to contain particularly high levels of EPA (eicosapentaenoic acid) and DHA (docosahexaenoic acid) [80]. Han et al. [80] reported that fermented fish oil contains DHA which can help alleviate the symptoms of atopic dermatitis. Fermented fish also contains natural antioxidants and essential nutrients [81]. It has been reported that bioactive peptides derived from fermented fish products act as antioxidants [81]. According to Siddegowda et al. [65], bioactive peptides may exert a variety of biological functions depending on their amino acid sequences, including antioxidant, antagonist, antihypertension, immunomodulatory, antithrombotic, antimicrobial, and anticancer activity. Aoshima and Ooshima [82] investigated the antioxidant activity of two Japanese liquid condiments, *shoyu* (soy sauce) and *gyoshoyu* (fish sauce). Siddegowda et al. [83] stated that the *Labeo rohita* head sauce produced by enzymatic and fermentation methods possesses in vitro antioxidant properties Antioxidants are critical for human health, as reactive oxygen species are thought to be a major cause of aging and carcinogenesis [84]. Siddegowda et al. [85] examined the sauce extracted from Rohu (*Labeo rohita*) enzymatically and fermentatively. They discovered that the total antioxidant activity (as ascorbic acid, g/ml), 2,2'-diphenyl-1-picryl-hydrazyl (DPPH, %), and 2,2'- azinobis-3-ethylbenzothizoline-6 sulfonate (ABTS, %) scavenging activities of fermented sauce samples were significantly higher at 3441± 60, 49.57 ± 1.7, and 99.0 ± 0.3, respectively, when compared with enzyme treated samples which showed 803 ± 13, 28.60 ± 1.1, and 78.4 ± 1.0, for the respective parameters, after 180 days storage at 37°C. Some research has suggested that fermented fish can produce bioactive metabolites (such as lactoferrin and flavonoids) as well as polysaccharides from raw materials [86]. These bioactive peptides are antioxidative [87], ACE inhibitors [88], antihypertensive [86] and have anti-proliferative effects on human lymphoma cells [89].

Biogenic amines have been reported from many fermented fish products and pose a safety concern [91]. These are nitrogenous compounds made up of amine groups formed by decarboxylation. Biogenic compounds are non-toxic when consumed in lower concentrations but can cause serious health implications when consumed in high amounts [92]. Biogenic amines are vasoactive compounds and may cause an increase in blood pressure, and people sensitive to these amines may show symptoms of allergy, headache, respiratory disorder, nausea, and tachycardia [93].The presence of elevated histamine and tyramine levels in 47 samples of fermented fish prepared from anchovy, squid, clam, and shrimp suggests that biogenic amine levels in fermented fish products should be closely monitored to ensure human health [94].

Biogenic amines (BAs) are a significant potential hazard in traditional fermented fish (*suan yu*), and *Enterobacteriaceae* are involved in their formation. Amine production capabilities of 97 *Enterobacteriaceae* strains isolated from traditional foods were examined [90]. The most significant physicochemical factor affecting amine production was pH. These bacteria are capable of adapting to the physicochemical conditions present during the product's fermentation process, posing significant safety risks. Pathogens like *Clostridium botulinum*, *Salmonella* sp., *Listeria* sp., and *Staphylococcus aureus* were also reported from many fermented fish products which may bear major health implications [95]. It is in such a context that the Cambodian government, as mentioned earlier, saw fit to enforce quality control standards on the nation’s representative fermented fish product, *prahok*.

## Cultural significance of fermented fish

This review revealed that certain foods have a lasting cultural significance. South and Southeast Asian countries have diverse and rich food cultures centering on, among other things, fermented food, in particular, fermented fish. However, though Asian foodways have found a global platform, fermented fish figures less prominently in the global food map, precisely because its strong flavor and odoriferous quality usually repel Western sensibilities and taste. There is considerable politics in deciding which food cultures are acceptable for global consumption [8]. Taste itself entails a high degree of cultural politics. Steffan Igor Ayora-Diaz argues that the symbolic aspects of food, and whether it is deemed fit to be consumed, depend on several cultural markers as religion, political and communal ideology, ethnicity, gender, and society as well as transnational factors like the UN and its policies on food security and food safety [96]. For example, in the very recent context of the COVID-19 pandemic, China and the Chinese have received a severe backlash (primarily from people in America and Europe) and regarded as being morally reprehensible due to their food cultures, simply because the practice of consuming bats and pangolins exists in some Chinese communities [96]. In the light of such factors, fermented fish, despite its strong local and overseas base of consumption, has remained relatively obscure in literary and cultural studies. For example, in the realm of the literature, though food is a central theme in Asian diasporic literature, fermented fish by itself is not a central presence even within this framework, partly because these novels have a huge Western readership unfamiliar with this food category. Again, while there is, admittedly, a greater awareness of the health benefits of fermented foods, the same has not translated to its greater inclusion in food guides in many countries around the world [97]. This is not because they are harmful, but rather due to the possibility that they are not part of the food cultures in a particular nation (and therefore unfamiliar) or they are too limited in their production and consumption to become a wider trend or tradition [97]. Thus, there is a significant gap between the relatively limited scientific exploration of ethnic food (including fermented fish) and the embeddedness of fermented fish in the literature and culture of the local communities. Even within food studies, the technical aspects for fermented fish production are more highlighted, with only a few (notable among them being Ruddle, Ishige, Grainger, Tamang and Yankowski) actually connecting it to a larger socio-cultural context. This warrants more extensive research on the interconnections between the scientific and the cultural aspects of fermented fish in Asian foodways.

Whereas fermented foods produced in Europe, North America, Australia, and New Zealand usually depend on defined starter cultures, those made in Asia and Africa often rely on spontaneous fermentation. Moreover, in developing countries, fermented foods are not often commercially produced on an industrial scale but rather in localized cottage industries. Although many fermented products rely on autochthonous microbes present in the raw material, for other products, the introduction of starter culture technology has led to greater consistency, safety, and quality. Microorganisms have already been shown to play an important role in making fermented fish more palatable and valuable. Furthermore, the nutrient composition and taste of the fish product also improve as a result of this process. However, many more traditional fermented fish products that have been consumed for centuries are yet to be thoroughly studied and characterized for their composition. Such products may have numerous isolates belonging to different species with undefined roles. Recent biotechnological tools have the potential to improve food fermentation through the development of novel starter cultures, as well as the addition of value through the fortification of nutrients and probiotics. This will heighten the health benefits associated with fermented food, at the same time promoting new businesses built around it. This, in turn, will assist the growth of cottage, small- and medium-sized businesses, which will be of particular import to Asian economies where there is a presence of significant poverty and economic disempowerment.

## Conclusion

This paper discussed the 18 different fermented fish products used in Southeast Asian cuisine, as well as their preparation method, nutrient composition, and cultural significance. The microbial profiles and nutrient composition reveal a diverse microbial population and good sources of probiotics, minerals, and other nutrients in these foods. The technology process of traditional fermented fish products is based on the substrate and source of the enzymes used in the fermentation, which comprises three groups, but the most common process is the fermentation which is caused by the enzymes found in fish. Fermented fish has been found to have especially high levels of EPA and DHA, as well as antioxidants and essential nutrients. Bioactive peptides derived from fermented fish products have been shown to have antioxidant, antagonist, antihypertension, and immunomodulatory, antithrombotic, antimicrobial, and anticancer activity. Fermented fish contained significantly more ascorbic acid, DPPH, and ABTS, which is beneficial, but certain bacteria produce biogenic amines, which may be poisonous if consumed in large quantities. As a result, monitoring the quality of fermented fish before consumption is critical. This study will be useful for future research into fermented fish products in order to highlight their long-term food value.

## Data Availability

All material/data used are available in the manuscript.
